# Prognostic factors of palatal mucoepidermoid carcinoma: a retrospective analysis based on a double-center study

**DOI:** 10.1038/srep43907

**Published:** 2017-03-06

**Authors:** Wenguang Xu, Yufeng Wang, Xiaofeng Qi, Junqi Xie, Zheng Wei, Xiteng Yin, Zhiyong Wang, Jian Meng, Wei Han

**Affiliations:** 1 Department of Oral and Maxillofacial Surgery, Nanjing Stomatological Hospital, Medical School of Nanjing University, No 30 Zhongyang Road, Nanjing, P.R. China; 2 Central Laboratory of Stomatology, Nanjing Stomatological Hospital, Medical School of Nanjing University, No 30 Zhongyang Road, Nanjing, P.R. China; 3 Department of Stomatology, Xuzhou Central Hospital, Affiliated Hospital of Medical College of Southeast University, Xuzhou 221009, People’s Republic of China

## Abstract

Mucoepidermoid carcinoma (MEC) of the palate is a common malignancy of minor salivary glands. This study was designed to identify the prognostic factors for MEC of the palate. The medical records of patients diagnosed with MEC of the palate who visited the Department of Oral and Maxillofacial Surgery at Nanjing Stomatological Hospital and the Department of Stomatology at Central Hospital of Xuzhou were retrospectively studied. The prognostic factors were determined using a Cox proportional hazards model. Furthermore, the expression of cancer stem cell (CSC) markers CD44, CD133, Nanog and Sox2 were detected in neoplastic samples of these patients by immunohistochemistry. As a result, both univariate analysis and multivariate analysis proved a high histological grade and an advanced tumor stage as negative prognostic factors for overall survival. By immunohistochemistry staining and survival analysis, a combination of CD44/CD133/SOX2 was found to have the strongest prognostic value for palatal MEC patients. In conclusion, the proposed nomogram which include histological grade and tumor stage along with cancer stem cell markers provides a more accurate long-term prediction for palatal MEC patients.

Mucoepidermoid carcinoma (MEC) is the most common malignancy of the salivary glands, accounting for 10–15% of all salivary gland neoplasms and 30% of all salivary malignancies^1^,^2^,^3^,^4^. To date, several authors have delineated the clinical and pathological features of and identified the prognostic factors for MEC of the salivary glands, particularly the major salivary glands, in studies including an adequate number of patients^2^,^5^,^6^,^7^,^8^. The prognostic factors for MEC of the salivary glands as a whole have been fully investigated. The histological grade, clinical stage, surgical margins, perineural and vascular involvement, and lymph node metastasis are the frequently identified prognostic factors for MECs^2^,^5^,^6^,^7^,^8^,^9^. However, due to the relative rarity of MEC of the palate, most researchers have reported single cases or studies with small sample sizes^9^,^10^,^11^,^12^,^13^. It remains inconclusive whether these clinicopathological prognostic factors identified for the MEC of major salivary gland are still effective and practicable for MEC of the palate. Apart from the clinical and pathological factors, potential biomarkers are also requisite for a more precise prognosis evaluation of MEC. Some markers, such as p53, ki-67, c-erbB-2, and CEA have been reported to be correlated with survival of patients with MEC^14^. In addition, a unique translocation t(11;19) (q21;p13), the most common genetic alteration in MECs, can produce a fusion oncogene known as CRTC1-MAML2^15^. Accumulating evidence revealed that the CRTC1-MAML2 expression correlates with a significantly better prognosis for MEC patients^16^,^17^,^18^,^19^.

Cancer stem cells (CSCs) are a small group of cells which are highly tumorigenic with the trend of self-renewal and potential to be differentiated into cells which composite the bulk of tumors. Notably, mounting evidence have proved that cancer stem cells play a major role in tumor progression, metastasis, recurrence, therapeutic resistance and ultimately the poor clinical outcome of patients in multiple types of cancers, including breast cancer, pancreatic cancer and head and neck cancer^20^,^21^,^22^. For MEC of salivary gland, cancer stem cells may also play a functional role in the pathobiology of the disease indicated by some studies^23^,^24^. Isolation and identification of cancer stem cells can be performed by protein markers which are differentially expressed between cancer stem cells and non-cancer stem cell population. The surface markers of cancer stem cells include CD133, CD44, Oct4, SOX2, Nanog, ALDH1, Bmi-1 and so on^25^. Recently, Maria and co-workers correlated the expression of several stem cell markers with histological and clinical parameters and found that Oct4 and Nanog correlated with perineural invasion in mucoepidermoid carcinoma tissues^26^. However, to the best of our knowledge, few studies directly validated the prognostic value of stem cell markers for patients with MEC. Moreover, further investigations are needed to select the appropriate cancer stem cell markers as possible prognostic factors for MEC patients.

From the perspectives, we designed the current study and investigated a relatively large cohort of patients with MEC of the palate from two major institutes to gain a more exhaustive understanding of the clinicopathological features and prognostic factors, with the aim of contributing to the establishment of guidelines for these lesions. Moreover, we attempted to evaluate the expression of cancer stem cell markers in neoplastic tissues to determine whether these markers have the potential to predict the outcomes and identify appropriate prognostic markers for patients with MEC of the palate.

## Results

### Patient demographics

A total of 75 patients diagnosed with MEC of the palate were eligible for inclusion, including 51 patients treated at the Department of Oral and Maxillofacial Surgery of Nanjing Stomatological Hospital and 24 patients at the Department of Stomatology of Central Hospital of Xuzhou. The mean and median age at diagnosis was 42.49 and 43 years old, respectively (range, 11–79 years). The women to men ratio was 2:1. The age and sex distribution of the included patients at the time of initial diagnosis was shown. The findings showed that MEC of the palate was more frequent in women in the fifth decade of life (Supplementary Figure S1).

The site of involvement was divided into the hard palate, junction of the hard and soft palate, and soft palate. The hard palate was the most frequent primary site (74.7%), followed by the junction of the hard and soft palate (22.7%). The most common presenting symptom was a painless mass in the palate (69.3%). Some patients also complained of non-healing oral ulceration in the palate at the time of diagnosis (14.7%). According to the TNM classification, T stage 1/2 tumors together accounted for most lesions (90.7%), while T stage 3/4 tumors accounted for the remaining minority (9.3%). All lesions (100%) exhibited N stage 0, with no lymphadenopathy on imaging and no palpable lymphadenopathy. None of the patients showed distant metastasis at the first time of diagnosis. Microscopically, 38 cases (50.7%) were classified as low grade, 31 cases (41.3%) as intermediate grade, and 6 cases (8.0%) as high grade of malignancy; only 7 cases (9.3%) showed positive nodal status, and the rest 68 cases (90.7%) exhibited negative nodal status. Of the total 75 patients, 60(80%) patients underwent surgery alone, 9 underwent surgery combined with radiotherapy, and 6 underwent surgery combined with radiotherapy and chemotherapy (Table 1).

### Survival analysis of clinical and pathological parameters for MEC of the palate

**Figure 1:**
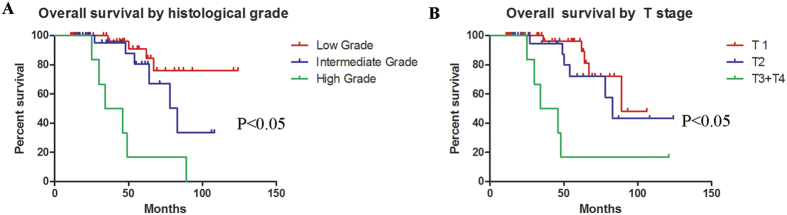
Overall survival estimated by histological grade (**A**) and tumor stage (**B**) for the 75 patients with MEC of the palate.

The clinical outcome data are presented in Table 2. The follow-up period ranged from 11 months to 124 months (median, 45 months). Kaplan–Meier analysis revealed that the 5-year and 10-year OS rates for patients with MEC of the palate were 77.3% and 44.0%, respectively. Univariate analysis identified a high histological grade (p = 0.001) and an advanced tumor stage (p = 0.001) as negative prognostic factors. Both factors resulted in a significant decrease in the 5-year OS rate (Fig. 1). On the other hand, the 5-year OS rate was not influenced by the following clinical characteristics: age, sex, presenting symptoms, tumor location and nodal status. Multivariate analysis also identified a high histological grade (HR, 3.421; 1.645–7.111; p = 0.001) and an advanced tumor stage (HR, 2.642; 1.251–5.578; p = 0.011) as significant prognostic factors.

**Figure 2:**
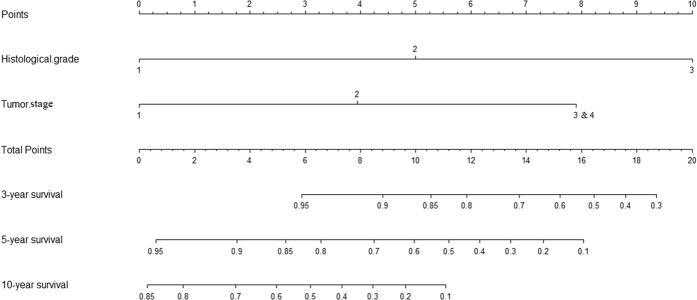
Nomogram including histological grade and tumor stage for predicting 3-year, 5-year and 10-year OS for palatal MEC patients.

The two risk factors, histological grade and tumor stage, which were statistically significant in multivariate analysis were introduced into the prognostic nomogram. (Fig. 2). The C-index of the established nomogram for predicting OS was 0.775. According to the total score identified on the points scale, the present nomogram can provide the likehood of 3-year, 5-year and 10-year OS for individual patients.

### Expressions of CD44, CD133, SOX2 and Nanog in MEC samples

**Figure 3:**
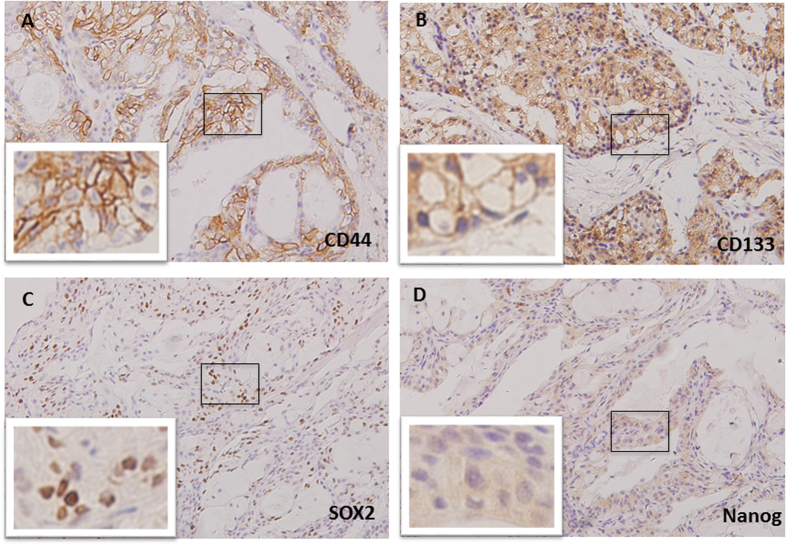
Immunohistochemical staining for CD44 (**A**), CD133 (**B**), SOX2 (**C**) and Nanog (**D**) in palatal MEC tissues.

The CSC markers CD44, CD133, SOX2 and Nanog protein expression was evaluated by immunohistochemical staining. Positive expressions of CD44 were mainly detected in the membrane, negative in 13 cases (17.3%), weakly positive in 34 cases (45.3%) and strongly positive in 28 cases (37.3%) (Fig. 3A). CD133-positive expression was mainly located in the membrane, negatively expressed in 12 cases (16.0%), weakly positively expressed in 29 (38.7%) and strongly positively expressed in 34 (45.3%) (Fig. 3B). SOX2 exhibited high expression in the nucleus of the tumor cells, with its expression being negative in 29 cases (38.7%), weakly positive in 27 (36.0%) and strongly positive in 19 (25.3%) (Fig. 3C). The Nanog expression was found both in the nucleus and cytoplasm of neoplastic cells, negatively expressed in 23 cases (30.7%), weakly expressed in 32 cases (42.7%), strongly expressed in 20 cases (26.6%) (Fig. 3D).

The expressions of CD44, CD133, SOX2 and Nanog were correlated with histological grade and tumor stage for MEC of the palate respectively (Table 3). However, no significant correlations were found between the above mentioned two clinicopathological factors along with the expression of CD44, CD133, SOX2 and Nanog. The inter-relationships among the expressions of CD44, CD133, SOX2 and Nanog was analyzed through Spearman’s rank correlation coefficient test (Table 4). The results showed that SOX2 was significantly correlated with Nanog (R = 0.363, p = 0.001). CD44 appeared to correlate with SOX2, while no statistically significant conclusion could be drawn (R = 0.215, p = 0.064).

### A combination of cancer stem cell markers improves the prediction of survival for palatal MEC patients

**Figure 4:**
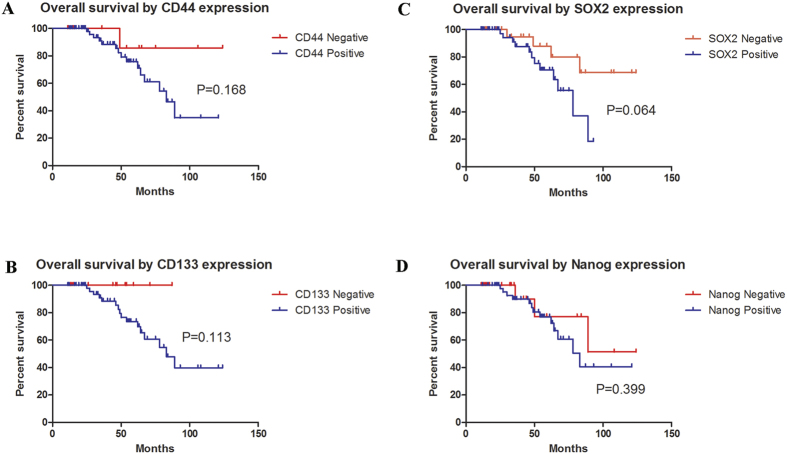
Kaplan–Meier curves estimated by the expression of CD44 (**A**), CD133 (**B**), SOX2 (**C**) and Nanog (**D**).

**Figure 5:**
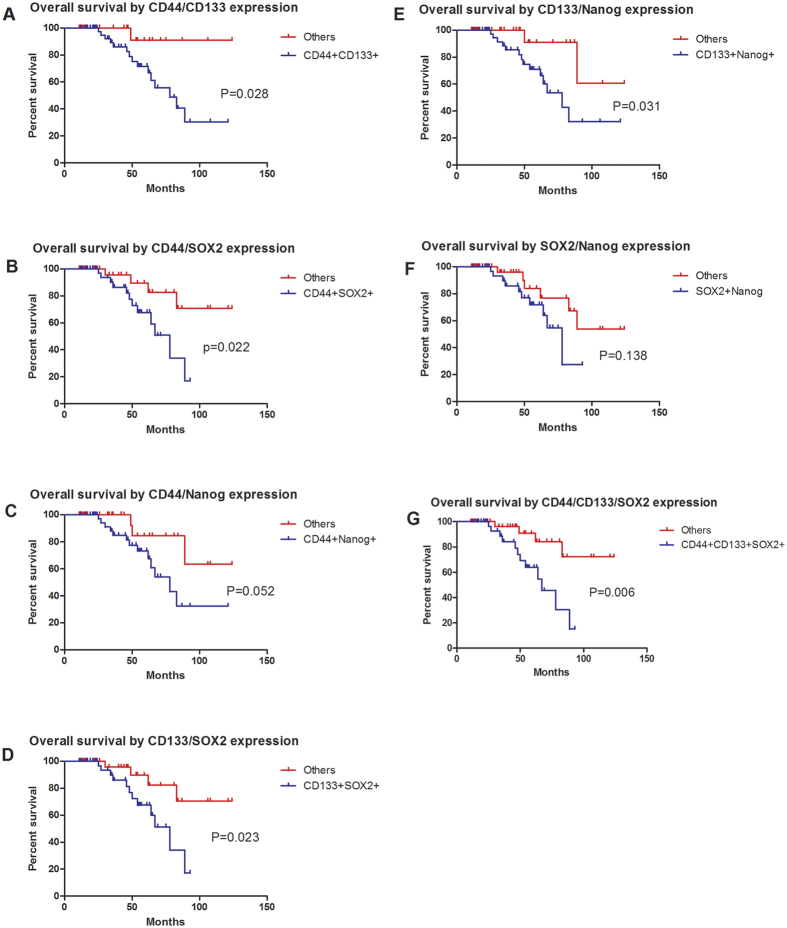
Kaplan–Meier curves estimated by various combination of markers, CD44/CD133 (**A**), CD44/SOX2 (**B**), CD44/Nanog (**C**), CD133/SOX2 (**D**), CD133/Nanog (**E**), SOX2/Nanog (**F**), CD44/CD133/Nanog (**G**).

**Figure 6:**
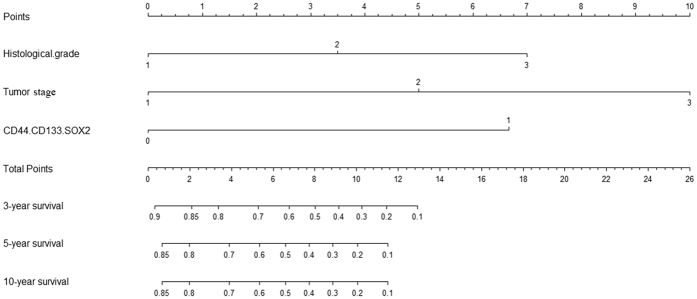
Nomogram including histological grade and tumor stage along with CD44/CD133/Nanog expression for predicting 3-year, 5-year and 10-year OS for palatal MEC patients.

The survival analysis concerning CD44, CD133, SOX2, Nanog was performed in the present series of MEC patients. The Kaplan–Meier analysis revealed that the expression levels of CD44 and CD133, SOX2 and Nanog were not significantly associated with their 5-year overall survival (P > 0.05) examined by log-rank test (Fig. 4), which indicated that these makers alone might not function as an effective prognosticator for MEC of the palate. We then investigated whether a combination of two or more cancer cell markers might provide more value in terms of prognostic value. Through an exhaustive permutation and combination of the four markers, as shown in Fig. 5, we found several combinations of markers had the potential to stratify the survival curves for MEC patients of the palate, including CD44/CD133, CD44/SOX2, CD133/SOX2, CD133/Nanog and CD44/CD133/SOX2, among which CD44/CD133/SOX2 having the strongest prognostic value. Multivariate analysis using the Cox proportional hazards model also revealed that CD44+/CD133+/SOX2+ expression was the independent negative prognostic factor for overall survival (HR, 5.105; 1.366–19.074; P = 0.015; Table 5). We next constructed a nomogram including histological grade, tumor grade and CD44/CD133/SOX2 expression levels (Fig. 6). The C-index of this nomogram for predicting OS was 0.851, which were significantly higher than that of the nomogram including only the histological grade and tumor grade (p < 0.05).

## Discussion

The clinicopathological prognostic factors for MEC of the salivary glands as a whole have been fully investigated. The histological grade, clinical stage, surgical margins, perineural and vascular involvement, and lymph node metastasis are the frequently identified prognostic factors for MECs^2^,^5^,^6^,^7^,^8^,^9^,^27^,^28^. The reported prognostic factors for MEC of the minor salivary glands were in line with those for the salivary glands as a whole^10^. In the present study, a high histological grade and an advanced tumor grade were determined as negative prognostic factors for overall survival, with the two risk factors identified as independent prognostic factors affecting survival in multivariate analysis.

Although various novel treatments have improved the quality of life of patients with salivary gland cancers, overall survival rates have remained low, especially in patients with advanced tumors^29^. Given that cancer stem cells may play a vital role in the pathogenesis and progression of various cancers, further understanding of the biology of these cells in the context of MEC may contribute to targeted treatments that benefit patients. In general, the cancer stem cell hypothesis has not been fully investigated in salivary gland tumors, especially in MEC. Previously, Adams *et al*. validated that salivary gland mucoepidermoid carcinomas contained a small population of cancer stem cells with enhanced tumorigenic potential in their generated cells lines and xenograft models, and these cancer stem cells were characterized by high ALDH activity and CD44 expression^23^,^30^. Their findings indicated that patients with MEC might benefit from therapies that obliterate these highly tumorigenic cells. Recently, Rodrigues *et al*. attempted to evaluate the expression of stem cell markers CD44, Bmi1, Oct4 and Nanog in non-neoplastic salivary gland tissue and MEC tissues, and correlate with clincopathological parameters^26^. As a result, Oct4 and Nanog was found correlated with perineural invasion in human salivary gland mucoepidermoid carcinoma. In the present study, we evaluated the expression of four cancer stem cell markers CD44, CD133, SOX2, Nanog in a large cohort of palatal MEC patients and identified some effective combinations of markers that have prognostic value for palatal MEC patients. A combination of CD44, CD133 and SOX2 was proved as a powerful and practicable prognosticator for patients with MEC of the palate.

CD44 is a commonly overexpressed cell marker in cancer cells and is widely recognized as a pivotal marker for the cancer stem cells, which confers highly malignant and therapeutic resistance properties^31^. Higher levels of CD44 expression was reported to be significantly correlated with malignant clinicopathological characteristics and worse prognosis of patients in multiple types of cancers. According to Benzion *et al*., a high frequency of cells expressing CD44 in head and neck cancer was correlated with tumor aggressiveness and poor prognosis^32^. In addition, some investigators demonstrated that CD44 had the potential to predict local recurrence of laryngeal cancer after radiotherapy^33^.

CD133, another widely accepted stem cell marker, is a cholesterol-binding membrane glycoprotein selectively correlated with plasma membrane protrusions^34^. According to Zhang *et al*., CD133(+) cells possessed higher clonogenicity and invasiveness, may lead to chemoresistance in oral squamous cell carcinoma^35^. In major salivary gland, CD133 is physiologically expressed at the apical membranes of secretory duct cells of major salivary glands. Karbanova and co-authors found that CD133 could be detected in both neoplastic and non-neoplastic salivary gland diseases^35^.

SOX2 is also an important cancer stem cell marker which plays a major role in the embryonic development and maintenance of stem cell pluripotency^36^,^37^. Increasing numbers of studies have suggested that SOX2 is involved in tumorigenesis and correlated with aggressive features in various types of malignancies^38^,^39^,^40^. The majority of studies indicated that SOX2 overexpression in cancer cells exhibited a deleterious outcome, and lead to relatively worse prognosis for patients^41^,^42^,^43^. However, several studies showed that increased levels of SOX2 was significantly associated with better prognosis for cancer patients^44^,^45^. In our study, the SOX2 expression alone appeared to have no relationship with prognosis for palatal MEC patients.

In the present series, CD44, CD133, SOX2 alone appeared to have little prognostic value for palatal MEC patients. This indicated that prognostic value of stem cell markers may depend on the specific types of cancers. However, CD44, CD133 and SOX2 combined together showed great prognostic performance. These meaningful results provide important clues to get a better understanding of how these markers may participate in cancer development and progression as well as highlight the possibility of identifying tumors biomarkers or future therapeutic targets.

Nevertheless, potential biases exist in our study. During the follow-up period of the study, no patients with distant metastasis or regional recurrence were found, partially because the disease had a relatively good prognosis, for another thing, the follow-up work may be not so perfect due to incomplete information collection. Moreover, some studies demonstrated that distant metastases or local recurrence were found even decades after the initial treatment of MEC of the major and minor salivary glands^10^,^46^. Therefore, a sufficient sample size and a long-term follow-up are crucial for determining the prognostic factors.

In addition, although some clinical parameters and CSC markers have been identified associated with prognosis of palatal MEC patients in our study, other biomarkers may also have prognostic value for the disease. The CRTC1-MAML2 fusion oncogene resulting from a recurring t(11;19) translocation is one of the star biomarkers^47^. A large proportional of related studies indicated that the CRTC1-MAML2 fusion oncogene defined a subgroup of MEC patients with favorable prognosis^16^,^17^,^18^,^19^. However, some other retrospective studies suggested that the CRTC1-MAML2 fusion status did not provide prognostic value^48^,^49^. In the present study, we also attempted to explore the association between CRTC1-MAML2 fusion and prognosis of palatal MEC patients. However, given that available histologic and frozen specimens are limited, we tentatively performed RT-PCR in six palatal MEC samples, and found that 5 out of 6 cases harbored CRTC1-MAML2 fusion oncogene (Supplementary Figure S2 and S3). Future prospective studies with large series are essential before any convictive conclusions can be drawn.

To the best of our knowledge, this is the first study that directly validates histological grade and tumor grade as the independent prognostic factors for patients with MEC of the palate in a relatively large cohort. More importantly, we have identified some combinations of cancer stem cell markers of important prognostic value, which significantly improves the prediction for overall survival for MEC patients of the palate. This will not only aid in patient counseling and contribute to a better understanding of the disease, but also will shed light on the escalation of therapeutic treatments of the disease.

## Materials and Methods

### Materials

The medical records of 75 patients diagnosed with MEC of the palate who visited the Department of Oral and Maxillofacial Surgery at Nanjing Stomatological Hospital and the Department of Stomatology at Central Hospital of Xuzhou between 2005 and 2015 were retrospectively studied. All experimental protocols in this study were performed in accordance with STROBE guidelines (Supplementary File S1) and were approved by the Ethics Review Board of the Nanjing Stomatological Hospital (approval number: 2015NL-008KS). The data was analyzed anonymously, and therefore no additional informed consent was required. We acquired data pertaining to the clinical and pathological features, treatment modalities, and follow-up, including sex, age at diagnosis, presenting symptoms, TNM stage, histological grade, nodal status, treatment modalities, clinical outcome, recurrence, and metastasis. A follow-up was conducted every 3 months. Patients who did not undergo treatment at the department were excluded. Two experienced doctors reclassified the disease using the TNM staging system according to the AJCC classification^50^. The histological grade for each tumor was evaluated and classified as low, intermediate, and high on the basis of the relative proportion of cell types as per Brandie’s classification^1^. The histological grade was agreed upon by two pathologists with no knowledge of the patients’ clinical outcomes, with specific focus on the tumor grade and nodal status. A third pathologist would intervene if agreements could not be reached between the two pathologists. Clinical and demographic data are summarized on Supplementary File S2.

### Immunohistochemistry (IHC)

Immunohistochemical analysis for CD44, CD133, Sox2 and Nanog proteins expression was performed in 75 MEC neoplastic samples tissue. Detailed immunohistochemistry protocol and antibodies dilutions are described in Supplementary File S3. Immunostaining evaluation was performed by two independent pathologists blinded to clinical data. The percentage of positive tumor cells was evaluated and graded as negative (0–10%), weakly positive (10–50%) and strongly positive (>50%), irrespective of cell type^26^,^42^. A third pathologist would intervene if agreements could not be reached between the two pathologists.

### RNA extraction and RT-PCR assay

Total RNA was extracted from frozen specimens of palatal MEC patients using TRIZOL Reagent (Invitrogen, Cat.No.15596-026) following their RNA isolation protocol. RNA was reverse transcribed into cDNA using a PrimeScript RT Master Mix kit (Takara, Code No. RR036A). One-tube RT-PCR followed by a nested PCR was carried out as described previously^16^. The amplification conditions consisted of a denaturation step at 95 °C for 3 minutes, followed by 35 cycles of 95 °C for 30 seconds, 55 °C for 30 seconds and 72 °C for 30 seconds. The program was completed by incubation at 72 °C for 5 minutes. The primers used for one-tube PCR were as follows: MECT1A 5′-AAGATCGCGCTGCACAATCA-3′ and MAML2A 5′-GGTCGCTTGCTGTTGGCAGG-3′. The first-round RT-PCR products were diluted with double distilled water to 1:50 and subjected to the following nested PCR. The amplification conditions of the second round are the same as that of the first round, and primer used were as follows: MECT1B 5′-GGAGGAGACGGCGGCCTT CG-3′ and MAML2B 5′-TTGCTGTTGGCAGGAGATAG-3′. The expected band size of the final PCR product was 117 bp, which was detected by gel electrophoresis (2–3% Metaphore agarose) and visualized with ethidium bromide staining under UV light. The PCR fragments obtained in the nested PCR were separated and purified. Then, sequencing was performed using ABI Big DyeTM v3.1 dye terminator cycle sequencing for CRTC1-MAML2 fusion positive MECs. The ubiquitously expressed β-actin mRNA fragment (190 bp) was amplified as an internal control for RNA quality.

### Statistical analysis

The correlations of CD44, CD133, Sox2 and Nanog expression and clinicopathological features were evaluated with Fisher’s exact test. The relationships among the expressions of CD44, CD133, SOX2 and Nanog were analyzed using Spearman’s rank correlation coefficient test and linear tendency test. The Kaplan–Meier product limit method was employed to generate overall survival (OS). Statistical significance was determined by log-rank tests. To evaluate the effects of prognostic factors on OS, univariate and multivariate hazard ratios (HRs) were determined using a Cox regression model. On multivariate regression analysis, a reduced Cox model was used. Based on all the independent prognostic factors, a nomogram was established by using the package of *rms* in R software version 3.2.3 (http://www.r-project.org/) for predicting 3-year, 5-year, 10-year overall survival. Harrell’s C-index was used in the nomogram for evaluating the discrimination. It can estimate the probability of concordance between the observed and predicted OS. The higher the C-index, the more precise was the survival prediction. All other statistical analyzes were performed using Statistical Product and Service Solutions software. A p-value of <0.05 was considered statistically significant. GraphPad Prism 5.0 software was used to create the figures.

## Additional Information

**How to cite this article**: Xu, W. *et al*. Prognostic factors of palatal mucoepidermoid carcinoma:a retrospective analysis based on a double-center study. *Sci. Rep.* **7**, 43907; doi: 10.1038/srep43907 (2017).

**Publisher's note:** Springer Nature remains neutral with regard to jurisdictional claims in published maps and institutional affiliations.

## Supplementary Material

Supplementary Information

## Figures and Tables

**Table 1:** Clinical and pathological characteristics of MEC of the palate.

Characteristics	No. (%) of patients
Age	
≤50	52 (69.3)
>50	23 (30.7)
Sex	
Male	25 (33.3)
Female	50 (66.7)
Tumor location	
Hard palate	56 (74.7)
Junction of hard and soft palate	17 (22.7)
Soft palate	2 (2.6)
Painless Mass	
Yes	52 (69.3)
No	23 (30.7)
Oral ulceration	
Yes	11 (14.7)
No	64 (85.3)
Tumor stage	
T1	41 (54.7)
T2	27 (36.0)
T3	6 (8.0)
T4	1 (1.3)
Nodal stage	
N0	75(100)
N1/N2	0
Metastatic stage	
M0	75 (100)
M1	0 (0)
Histological grade	
Low Grade	38 (50.7)
Intermediate Grade	31 (41.3)
High Grade	6 (8.0)
Nodal status	
Positive	7 (9.3)
Negative	68 (90.7)
Treatment	
Surgery alone	60 (80.4)
Surgery + Radiotherapy	9 (13.7)
Surgery + Radiotherapy + Chemotherapy	6 (5.9)

**Table 2:** Survival analysis of clinical and histopathological variables for MEC of the palate.

Variables	Univariate analysis		Multivariate analysis	
	5-year OS (%)	p	p	HR (95% CI)
Age				
≤50	81.3			
>50	65.9	0.967		
Gender				
Male	74.0			
Female	78.9	0.911		
Tumor location				
Hard palate	70.3			
Junction of hard and soft palate & soft palate	100	0.073		
Tumor stage				
Stage 1	96.0			
Stage 2	71.9			
Stage 3 and 4	16.7	0.001	0.011	2.642 (1.251–5.578)
Histological grade				
Low Grade	93.3			
Intermediate Grade	79.7			
High Grade	33.3	0.001	0.001	3.421 (1.645–7.111)
Nodal status				
Positive	66.7			
Negative	78.0	0.230		
Ulceration				
Yes	66.7			
No	83.9	0.435		
Smoking				
Yes	100			
No	58.7	0.499		

Abbreviations: OS, overall survival; HR, hazards ratio; CI, confidence interval; R, radiotherapy; C, chemotherapy.

**Table 3:** Correlation of CD44, CD133, SOX2 and Nanog expression with clinicopathological factors for MEC of the palate.

Variables	CD44		P-value	CD133		P-value	SOX2		P-value	Nanog		P-value
	−	+/++		−	+/++		−	+/++		−	+/++	
Histological grade												
Low grade	7	31		8	30		17	21		13	25	
Intermediate grade	5	26		4	27		10	21		9	22	
High grade	1	5	0.829	0	6	0.556	2	4	0.592	1	5	0.803
Tumor stage												
T1	6	35		5	36		13	28		14	27	
T2	6	21		7	20		13	14		8	19	
T3 & T4	1	6	0.807	0	7	0.214	3	4	0.387	1	6	0.663

**Table 4:** Relationships among the expressions of CD44, CD133, SOX2 and Nanog.

Markers	CD44	CD133	SOX2	Nanog
CD44	1			
CD133	R = −0.008, p = 0.948	1		
SOX2	R = 0.215, p = 0.064	R = 0.176, p = 0.130	1	
Nanog	R = 0.001, p = 0.993	R = 0.183, p = 0.116	R = 0.363, p = 0.001	1

**Table 5:** Multivariate analysis of 75 palatal MEC patients.

Variables	Multivariate analysis	
	p	HR (95% CI)
Histological grade	0.002	2.354 (1.100–5.034)
Tumor stage	0.027	3.397 (1.584–7.286)
CD44/CD133/SOX2	0.015	5.105 (1.366–19.074)
