# Supplementary material for: Prognostic factors of palatal mucoepidermoid carcinoma: a retrospective analysis based on a double-center study
Source: Sci Rep. 2017 Mar 6;7:43907. doi: 10.1038/srep43907 (PMC5338264; doi:10.1038/srep43907)
Supplement: Supplementary Information [file srep43907-s1.pdf]

Prognostic factors of palatal mucoepidermoid carcinoma: a retrospective analysis based on a double-center study

Xu Wenguang[1,2], Wang Yufeng[1,2], Qi Xiaofeng[1,2], Xie Junqi[1,2], Wei Zheng[1,2], Yin Xiteng[1,2], Wang Zhiyong[1,2], Meng Jian[3], Han Wei[1,2*]

1. Department of Oral and Maxillofacial Surgery, Nanjing Stomatological Hospital, Medical School of Nanjing University, No 30 Zhongyang Road, Nanjing, P.R China

2. Central Laboratory of Stomatology, Nanjing Stomatological Hospital, Medical School of Nanjing University, No 30 Zhongyang Road, Nanjing, P.R China

3. Department of Stomatology, Xuzhou Central Hospital, Affiliated Hospital of Medical College of Southeast University, Xuzhou 221009, People's Republic of China.

The first two authors contribute equally to this paper.

***Corresponding author: Han Wei**

Email: doctorhanwei@hotmail.com

Tel: +86 25 83620140

Fax: +86 25 83620173

Supplementary Figure S1. Age and sex distribution for the 75 patients with mucoepidermoid carcinoma (MEC) of the palate

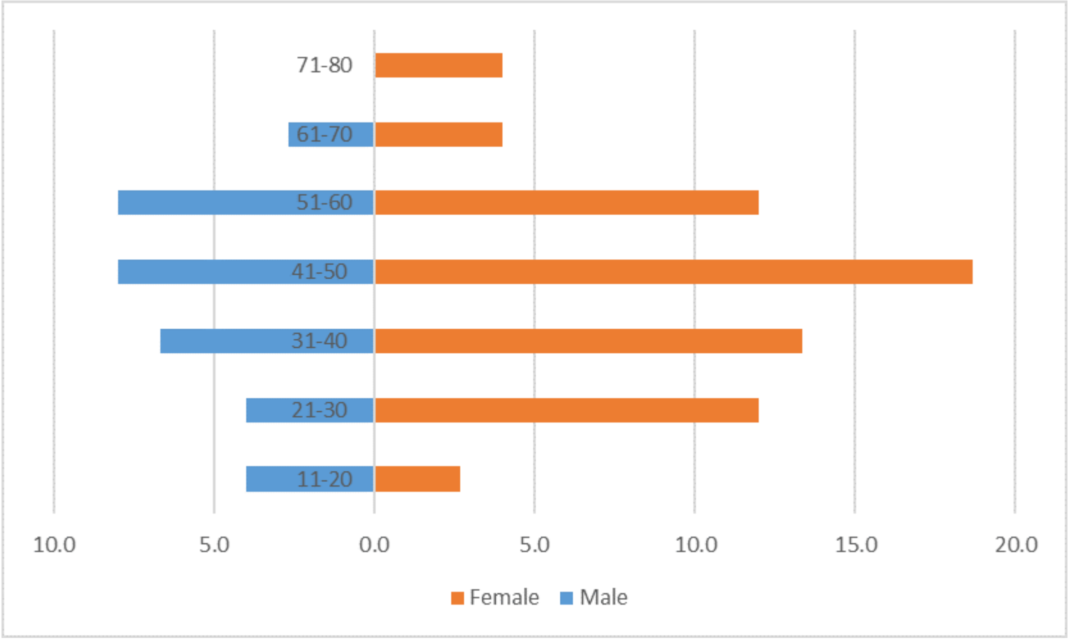

Supplementary Figure S2. Detection of the CRTC1-MAML2 fusion transcript by RT-PCR Mucoepidermoid carcinoma cases CRTC1-MAML2 fusion positive (lanes1-3 and 5-6) and negative (lane 4).

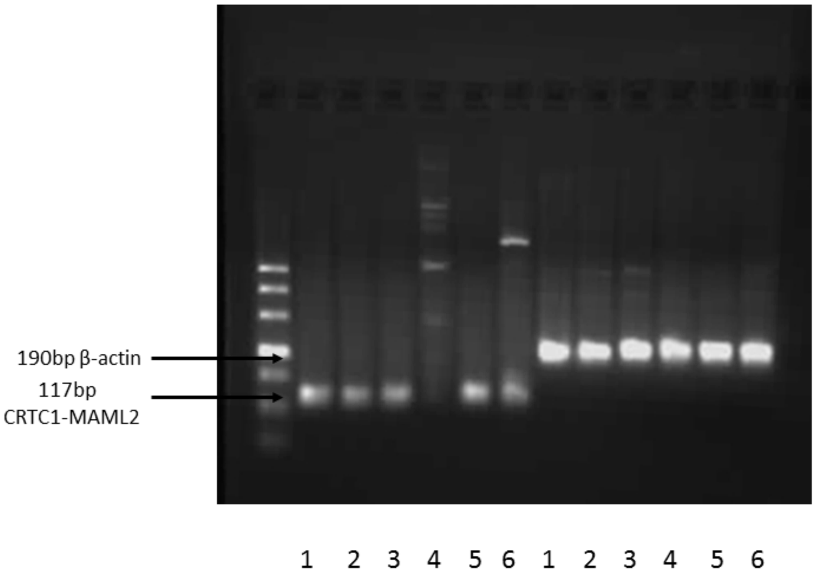

Supplementary Figure S3. Sequence analysis of MECT1-MAML2 fusion positive samples. Nucleotide sequence analysis of the PCR-product generated from the palatal MECs validated that it corresponded to a chimeric transcript in which exon 1 of CRTC1 is linked to exon 2 of MAML2.

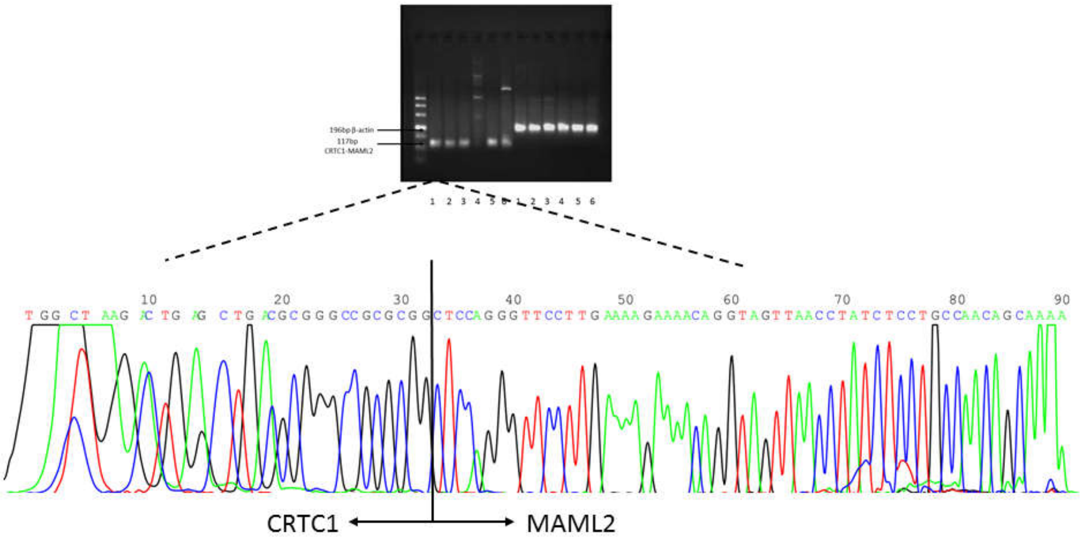

Supplementary File S1: STROBE checklist cohort of the article

STROBE Statement—Checklist of items that should be included in reports of ***cohort studies***

	Item No	Recommendation	Reported on page #
**Title and abstract**	1	(*a*) Indicate the study's design with a commonly used term in the title or the abstract	1
		(*b*) Provide in the abstract an informative and balanced summary of what was done and what was found	2
**Introduction**			
Background/rationale	2	Explain the scientific background and rationale for the investigation being reported	3&4
Objectives	3	State specific objectives, including any prespecified hypotheses	4&5
**Methods**			
Study design	4	Present key elements of study design early in the paper	12
Setting	5	Describe the setting, locations, and relevant dates, including periods of recruitment, exposure, follow-up, and data collection	12 and Supplementary File 2
Participants	6	(*a*) Give the eligibility criteria, and the sources and methods of selection of participants. Describe methods of follow-up	12
		(*b*) For matched studies, give matching criteria and number of exposed and unexposed	NA
Variables	7	Clearly define all outcomes, exposures, predictors, potential confounders, and effect modifiers. Give diagnostic criteria, if applicable	12&13
Data sources/ measurement	8*	For each variable of interest, give sources of data and details of methods of assessment (measurement). Describe comparability of assessment methods if there is more than one group	12 and Supplementary File 2
Bias	9	Describe any efforts to address potential sources of bias	13
Study size	10	Explain how the study size was arrived at	12
Quantitative variables	11	Explain how quantitative variables were handled in the analyses. If applicable, describe which groupings were chosen and why	13
Statistical methods	12	(*a*) Describe all statistical methods, including those used to control for confounding	13,14
		(*b*) Describe any methods used to examine subgroups and interactions	NA
		(*c*) Explain how missing data were addressed	13
		(*d*) If applicable, explain how loss to follow-up was addressed	13
		(*e*) Describe any sensitivity analyses	NA
**Results**			
Participants	13*	(a) Report numbers of individuals at each stage of study—eg numbers potentially eligible, examined for eligibility, confirmed eligible, included in the study, completing follow-up, and analysed	5
		(b) Give reasons for non-participation at each stage	NA
		(c) Consider use of a flow diagram	NA
Descriptive data	14*	(a) Give characteristics of study participants (eg demographic, clinical, social) and information on exposures and potential	5

		confounders	
		(b) Indicate number of participants with missing data for each variable of interest	6
		(c) Summarise follow-up time (eg, average and total amount)	6
Outcome data	15*	Report numbers of outcome events or summary measures over time	6
Main results	16	(*a*) Give unadjusted estimates and, if applicable, confounder-adjusted estimates and their precision (eg, 95% confidence interval). Make clear which confounders were adjusted for and why they were included	6-8
		(*b*) Report category boundaries when continuous variables were categorized	6–8
		(*c*) If relevant, consider translating estimates of relative risk into absolute risk for a meaningful time period	6-8
Other analyses	17	Report other analyses done—eg analyses of subgroups and interactions, and sensitivity analyses	NA
**Discussion**			
Key results	18	Summarise key results with reference to study objectives	9
Limitations	19	Discuss limitations of the study, taking into account sources of potential bias or imprecision. Discuss both direction and magnitude of any potential bias	11
Interpretation	20	Give a cautious overall interpretation of results considering objectives, limitations, multiplicity of analyses, results from similar studies, and other relevant evidence	10
Generalisability	21	Discuss the generalisability (external validity) of the study results	11
**Other information**			
Funding	22	Give the source of funding and the role of the funders for the present study and, if applicable, for the original study on which the present article is based	16

*Give information separately for exposed and unexposed groups.

Note: An Explanation and Elaboration article discusses each checklist item and gives methodological background and published examples of transparent reporting. The STROBE checklist is best used in conjunction with this article (freely available on the Web sites of PLoS Medicine at http://www.plosmedicine.org/, Annals of Internal Medicine at http://www.annals.org/, and Epidemiology at http://www.epidem.com/). Information on the STROBE Initiative is available at http://www.strobe-statement.org.

Supplementary File S2. Raw data of 75 palatal MEC patients

ID	Sex	Age	Survival	Outcome	Radiother	Chemother	Treatment modal
60	1	43	47	0	0	0	1
68	2	65	44	0	0	0	1
40	2	42	124	0	0	0	1
57	2	34	11	0	0	0	1
21	2	18	37	0	0	0	1
23	1	34	33	0	0	0	1
31	2	52	17	0	0	0	1
1	2	79	106	0	0	0	1
19	1	21	42	0	0	0	1
55	2	47	24	0	0	0	1
50	2	69	30	1	1	1	3
51	2	45	27	1	0	0	1
11	1	21	61	0	0	0	1
37	1	48	15	0	0	0	1
71	2	25	54	0	0	0	1
4	2	40	71	0	1	0	2
26	2	43	26	0	0	0	1
33	2	47	14	0	0	0	1
38	2	24	13	0	0	0	1
53	2	24	59	0	1	0	1
62	2	39	46	0	0	0	1
66	1	49	53	0	0	0	1
73	2	46	87	0	0	0	1
9	1	20	63	0	0	0	1
35	2	57	16	0	1	0	2
65	1	19	24	0	1	0	2
72	2	36	24	0	0	0	1
16	2	42	49	1	0	0	1
6	2	24	67	0	0	0	1
24	1	70	32	0	0	0	1
28	2	57	21	0	1	0	2
34	2	52	17	0	0	0	1
41	2	40	121	0	0	0	1
58	1	45	16	0	0	0	1
75	1	48	21	0	0	0	1
15	2	45	50	1	0	0	1
44	2	31	89	1	1	0	2
52	1	42	34	1	1	1	3
10	1	34	63	0	0	0	1
29	1	22	19	0	0	0	1
32	2	48	17	0	1	1	3
36	2	46	16	0	0	0	1
61	1	52	35	0	1	0	2
64	2	29	57	0	0	0	1

67	2	53	22	0	0	0	1
70	1	67	35	0	0	0	1
17	1	31	48	1	0	0	1
7	2	25	65	0	0	0	1
22	2	42	36	0	0	0	1
56	1	53	75	0	0	0	1
2	2	32	84	0	0	0	1
3	1	57	81	0	0	0	1
18	2	76	45	0	0	0	1
25	1	56	27	0	0	0	1
39	2	24	12	0	0	0	1
42	1	11	108	0	1	0	2
54	2	16	21	0	0	0	1
46	2	24	78	1	0	0	1
49	2	51	54	1	0	0	1
69	1	53	25	1	1	1	3
5	2	44	69	0	0	0	1
8	2	52	63	0	1	0	2
12	1	54	56	0	0	0	1
13	1	39	55	0	1	1	3
14	2	49	50	0	0	0	1
20	2	59	40	0	1	0	2
27	2	68	23	0	0	0	1
30	2	24	19	0	0	0	1
43	2	56	93	0	0	0	1
45	2	31	83	1	0	0	1
47	2	36	64	1	0	0	1
48	2	36	62	1	0	0	1
59	2	75	46	1	1	0	3
63	2	42	67	1	0	0	1
74	1	37	36	1	0	0	1

Histological	Tumor gra	Nodal sta	Mass	Ulceratic	Tumor loc	Smoking	CD44 score
1	1	0	1	0	1	0	1
1	1	1	1	0	2	0	1
1	2	0	1	0	2	0	0
1	1	0	2	0	1	0	0
1	1	0	2	0	1	0	2
1	1	0	1	0	1	1	2
1	1	0	2	1	1	0	2
2	1	0	1	1	1	0	0
1	1	0	1	0	1	0	1
2	2	1	1	0	1	0	1
3	3	0	2	0	1	0	1
2	2	0	2	0	1	0	1
2	1	0	2	0	2	0	2
1	2	0	1	0	1	1	0
2	2	0	1	0	1	0	0
2	2	0	1	0	1	0	1
1	2	0	1	0	1	0	1
2	1	0	2	0	1	0	1
1	1	0	1	0	1	0	1
2	2	0	1	0	2	0	1
1	2	0	1	1	1	0	1
1	1	0	1	0	1	0	1
1	2	0	1	1	1	0	1
1	2	0	1	0	2	0	0
2	2	1	1	0	2	0	0
2	4	0	2	1	1	0	0
1	1	1	2	0	2	1	0
3	2	0	2	1	1	0	0
1	1	0	1	0	3	0	1
2	1	0	2	1	2	0	1
2	1	0	2	0	1	0	1
2	1	0	1	0	2	0	1
1	3	0	1	0	1	0	1
1	2	0	2	0	1	0	1
2	1	0	1	0	1	0	1
1	2	0	1	0	1	0	1
3	1	0	1	0	2	0	1
3	3	0	2	1	1	0	1
1	1	0	2	0	1	1	2
1	2	0	1	1	1	0	2
2	2	0	2	0	1	0	2
2	2	0	1	0	2	0	2
2	1	0	2	0	1	0	2
1	1	0	1	0	1	0	2

1	1	0	1	0	1			0	2
1	2	0	1	0		1	1		2
2	3	0	1	0	1		0		2
1	1	0	2	0	2		0		0
2	1	0	1	0	3		0		0
1	1	0	1	0	1		0		0
1	1	0	1	0	1		0		1
1	1	0	1	0	1		0		1
2	2	0	1	0	1		0		1
2	1	0	1	0	2		0		1
1	1	0	1	0	1		0		1
2	2	0	1	0	1		0		1
1	2	0	2	0	1		1		1
2	2	0	1	0	1		0		1
2	2	0	2	0	1		0		1
3	3	0	1	1		1	0		1
1	1	0	2	0	2		0		2
2	1	0	1	0	1		0		2
1	1	0	1	0	1		0		2
2	1	1	2	0	2		0		2
1	2	0	1	0	1		0		2
2	2	0	1	1	1		0		2
2	1	0	1	0	2		0		2
2	1	0	1	0	2		0		2
1	1	0	1	0	1		0		2
2	2	0	2	0	1			0	2
2	1	0	1	0	1		0		2
1	1	0	1	0	1		0		2
3	3	1	1	0	1		0		2
1	1	1	1	0	1		0		2
1	1	0	1	0		1	0		2

CD44 expres	CD133 score	CD133 expre	SOX2 score	SOX2 expres	Nanog score	Nanog expre
0	0	0	1	0	1	0
0	0	1	2	0	0	0
0	1	1	0	1	0	1
0	1	1	1	0	0	1
0	1	0	1	0	1	1
0	1	1	0	0	0	1
0	1	1	0	0	0	1
0	2	1	0	0	1	1
0	2	1	0	1	0	1
0	2	1	2	1	2	1
0	2	1	0	1	1	1
0	2	1	1	1	1	0
0	2	1	2	0	2	0
1	0	1	0	1	0	1
1	0	1	0	1	1	1
1	0	1	2	0	1	0
1	0	1	0	1	0	1
1	0	1	0	1	0	1
1	0	1	1	0	0	0
1	0	0	0	0	0	0
1	0	0	0	1	1	0
1	0	1	1	1	1	1
1	0	1	0	0	1	1
1	1	1	0	0	1	0
1	1	1	2	1	2	1
1	1	1	0	0	0	1
1	1	1	0	1	1	1
1	1	1	0	1	1	1
1	1	1	1	0	1	1
1	1	1	1	0	0	0
1	1	0	0	0	0	0
1	1	1	0	0	0	0
1	1	1	0	0	1	0
1	1	1	2	1	1	1
1	1	1	1	0	2	0
1	1	1	1	1	0	1
1	1	0	1	0	0	0
1	1	1	1	1	1	1
1	1	1	0	1	2	1
1	1	1	0	1	0	1
1	1	1	2	1	0	1
1	1	1	1	1	1	0
1	1	1	1	1	0	1
1	1	1	1	1	1	1

1	1	1	0	0	2	1
1	1	1	1	1	1	1
1	1	1	2	0	2	1
1	2	1	2	0	1	0
1	2	0	2	1	2	1
1	2	1	2	1	1	1
1	2	1	0	1	0	1
1	2	1	0	1	0	1
1	2	1	2	1	1	1
1	2	1	1	1	1	0
1	2	1	1	1	1	1
1	2	1	0	1	0	0
1	2	1	2	0	2	0
1	2	1	1	1	2	1
1	2	1	1	1	1	1
1	2	0	1	1	1	1
1	2	1	2	1	1	0
1	2	0	1	0	2	1
1	2	0	1	1	1	1
1	2	1	1	0	2	1
1	2	0	1	1	2	0
1	2	1	0	1	2	1
1	2	0	2	0	2	1
1	2	1	2	1	2	0
1	2	1	1	0	1	1
1	2	1	0	1	2	1
1	2	1	2	1	1	1
1	2	1	0	1	2	1
1	2	1	2	1	2	1
1	2	1	2	1	2	1
1	2	1	1	1	1	1

CD44CD133SOX2
0
0
0
0
1
0
0
0
0
1
0
1
1
0
0
0
0
0
0
0
0
0
0
0
0
0
0
0
1
1
0
0
0
1
1
1
1
1
0
0
1
1
1
1

0
1
1
0
0
0
0
0
1
1
1
0
1
1
1
1
1
1
1
1
1
0
1
1
1
0
1
0
1
1
1

Supplementary File S3. Immunohistochemistry protocol and antibodies dilutions

Four-µm-thick tissue sections were cut, deparaffinised, and subjected to antigen recovery treatment with 100 mM citrate buffer target retrieval solution, pH 6.0 at 95 ºC, in a water bath for 20 minutes. Endogenous peroxidase activity was blocked by incubating with phosphate-buffered saline (PBS) and 3% hydrogen peroxidase for 30 minutes. After washing with PBS, the sections were incubated with monoclonal rabbit anti-CD44 (1:400, ab51037, Abcam), polyclonal rabbit anti-CD133 (1:100, ab19898, Abcam), monoclonal rabbit anti-Sox2 (1:200, ab92494, Abcam), and monoclonal rabbit anti-Nanog (1:100, ab109250, Abcam) and overnight, followed by the Envision Dual Link System HRP method (Dako, K4061) for CD44, CD133, and Sox2 and Nanog antibodies. All antibodies were diluted in Dako antibody diluent. Reactions were revealed by incubating the sections with 3,3'-diaminobenzidine tetrahydrochloride (Dako, K3468). The negative controls were obtained by substituting the primary antibody with non-immune serum. CD44 and CD133 positivity was membranous, Sox2 positivity was nuclear and Nanog expression was considered as both nuclear and cytoplasmic staining. As positive controls for CD44, CD133 and Sox2 and Nanog, normal oral mucosa tissues were used.
